# Cotard Syndrome without Depressive Symptoms in a Schizophrenic Patient

**DOI:** 10.1155/2015/643191

**Published:** 2015-05-25

**Authors:** Pedro Morgado, Ricardo Ribeiro, João J. Cerqueira

**Affiliations:** ^1^Life and Health Sciences Research Institute (ICVS), School of Health Sciences, University of Minho, Braga, Portugal; ^2^ICVS-3Bs PT Government Associate Laboratory, Braga/Guimaraes, Portugal; ^3^Hospital de Braga, Braga, Portugal

## Abstract

*Introduction*. Cotard syndrome is a rare condition characterized by nihilistic delusions concerning body or life that can be found in several neuropsychiatry conditions. It is typically associated with depressive symptoms. *Method*. We present a case of Cotard syndrome without depressive symptoms in the context of known paranoid schizophrenia. A literature review of Cotard syndrome in schizophrenia was performed. *Results*. Although there are few descriptions of this syndrome in schizophrenia, patients usually present depressive mood and psychomotor retardation, features not seen in our patient. Loss of the sense of the inner self, present in schizophrenia, could explain patient's symptomatology but neurobiological bases of this syndrome remain unclear. *Conclusion*. Despite not being considered in actual classifications, Cotard syndrome is still relevant and psychiatric evaluation is critical to diagnosing and treating this condition in psychiatric patients.

## 1. Introduction

Cotard syndrome (CS) has been described by Jules Cotard (1880) as a nihilistic delusion, which may range from negation of existence of parts of the body to delusion of being dead and negation of self-existence. This syndrome can be found in numerous psychiatric or neurologic conditions, such as mood disorders, schizophrenia and other psychotic disorders, dissociative disorders, central nervous system (CNS) infections, cerebrovascular disease, CNS neoplasm, and traumatic brain injury [1–3]. CS typically affects middle-aged or older people and the typical picture also includes depressive mood, which is present in approximately 90% of cases described in the literature [1]. Presentation at younger ages has been described as being associated with an increased risk of bipolar disorder [2].

We report on a patient with known schizophrenia and history of opioid abuse that developed a CS one year after medication dropout. To our knowledge, only few cases have been described reporting CS without depressive symptoms.

## 2. Case Report

Our patient was a 42-year-old male who was treated for paranoid schizophrenia at our psychiatric department for 10 years. He had also a history of opioid abuse, being abstinent for 4 years. He was medicated with haloperidol decanoate 100 mg intramuscularly (i.m.) every 4 weeks but discontinued treatment one year after the current admission.

He was brought to the emergency room (ER) by his cousin for behavioural alterations and paranoid speech. Progressively over a 2-month period he had become more isolated and developed paranoid thoughts, reporting that someone had captured him and controlled his behavior and thoughts. He also showed nihilistic delusions concerning his body (“my heart does not beat,” “my stomach are being destroyed and I'm being fed by tube,” “my palate disappeared” and “I have no blood”) and stated he was going to die that day. He presented restricted affect but psychomotor activity was enhanced and mood was euthymic. Physical examination was normal. Laboratory assessment (complete blood count, electrolytes, creatinine levels, and thyroid hormones) was within normal values. Urine drug screen test was negative and blood ethanol levels were undetectable. Brain CT did not demonstrate any pathology.

He was admitted to our inpatient psychiatric unit and medicated with paliperidone 12 mg/d and lorazepam 5 mg/d. As the patient was initially reluctant to take medication, haloperidol 10–15 mg/d was administered intramuscularly. Due to the extrapyramidal side effects of haloperidol, biperiden was added, 4 mg/d. After one week of treatment, he started to improve his behavior and delusional ideation became less intense. The patient was discharged 24 days after admission in a state of full remission, as paranoid and nihilistic delusions completely disappeared. After discharge he maintained treatment with paliperidone 12 mg/d and during the 6-month follow-up no somatic or psychotic symptoms were reported by the patient or detected during clinical evaluations. Thus, the diagnosis of paranoid schizophrenia was maintained.

## 3. Discussion 

In this case report, we present an uncommon form of CS as the patient did not display any depressive symptoms associated with his nihilistic delusions. The described patient suffered from an exacerbation of his known paranoid schizophrenia, which responded to monotherapy with an atypical antipsychotic, achieving full remission of delusions.

In fact, CS is typically associated with depressive mood and psychomotor inhibition [1] and treatment usually involves antidepressants, mood stabilizers, and/or electroconvulsive therapy (ECT) [3]. Even in the context of mood disorders, this syndrome is rare. A review of 479 psychiatric patients admitted to inpatient treatment in Mexico, including 150 suffering from schizophrenia, identified 3 cases of CS (0.62%) but none of them in the context of schizophrenia [4]. Despite being well known that nihilistic delusions can occur in schizophrenia, only few single cases or small series report this occurrence [5–9]. In a recent review of 346 cases of schizophrenia, CS could be diagnosed in three cases (0.87%), all associated with loss of energy [10]. Interestingly, this contrasts with enhanced psychomotor activity observed in our patient.

Apart from restricted affect, our patient did not display any other alteration of mood and emotions. Indeed, affective flattening and abnormal affective processing, mediated by dysfunctional flow of information from sensory cortices to the limbic system, were suggested to be related with monothematic delusions in schizophrenia [11]. In addition, abnormalities in nondominant frontal, temporal, and parietal lobes, areas known to be affected by schizophrenia, have also been related with CS [3].

Some authors considered nihilist delusions as an extreme manifestation of the topic of death [10]. However, considering the cooccurrence of Cotard and Capgras syndromes (the latter is the most common misidentification syndrome) [5, 7, 12, 13], it could be conceptualized that both monothematic and bizarre delusions are related with loss of the sense of the inner self that is core and typical of schizophrenia. Alternately, CS can be conceptualized as a dysfunction of egocentric mind-representing system (as asomatognosia is conceptualized as damage of the egocentric body-representing system) [14]. This phenomenon can be mediated by dysfunction of insular cortex, an area that plays a central role in the conscious awareness of internal sensations and that is known to be affected by stress [15, 16].

Although CS is not a psychiatric diagnosis but a feature of several neurological and psychiatric conditions, psychiatrists should be aware of this uncommon psychotic phenomenon to prompt diagnosing, describing, and treating according to the best evidence.

## 4. Conclusion

Despite not being considered in actual classifications, Cotard syndrome is still relevant and clinical recognition is critical to diagnosing and treating this condition in psychiatric patients.
